# Supplementary material for: Subcellular organization of viral particles during maturation of nucleus-forming jumbo phage
Source: Sci Adv. 2022 May 4;8(18):eabj9670. doi: 10.1126/sciadv.abj9670 (PMC9067925; doi:10.1126/sciadv.abj9670)
Supplement: Supplementary file 1 — Figs. S1 to S7 Table S1 [file sciadv.abj9670_sm.pdf]

Science Advances
AAAS

Supplementary Materials for

Subcellular organization of viral particles during maturation of nucleus-forming jumbo phage

Vorrapon Chaikeeratisak, Kanika Khanna, Katrina T. Nguyen, MacKennon E. Egan, Eray Enustun, Emily Armbruster, Jina Lee, Kit Pogliano, Elizabeth Villa,* Joe Pogliano*

*Corresponding author. Email: jpogliano@ucsd.edu (J.P.); evilla@ucsd.edu (E.V.)

Published 4 May 2022, *Sci. Adv.* **8**, eabj9670 (2022)
DOI: 10.1126/sciadv.abj9670

The PDF file includes:

Figs. S1 to S7
Table S1
Legends for movies S1 to S4

Other Supplementary Material for this manuscript includes the following:

Movies S1 to S4

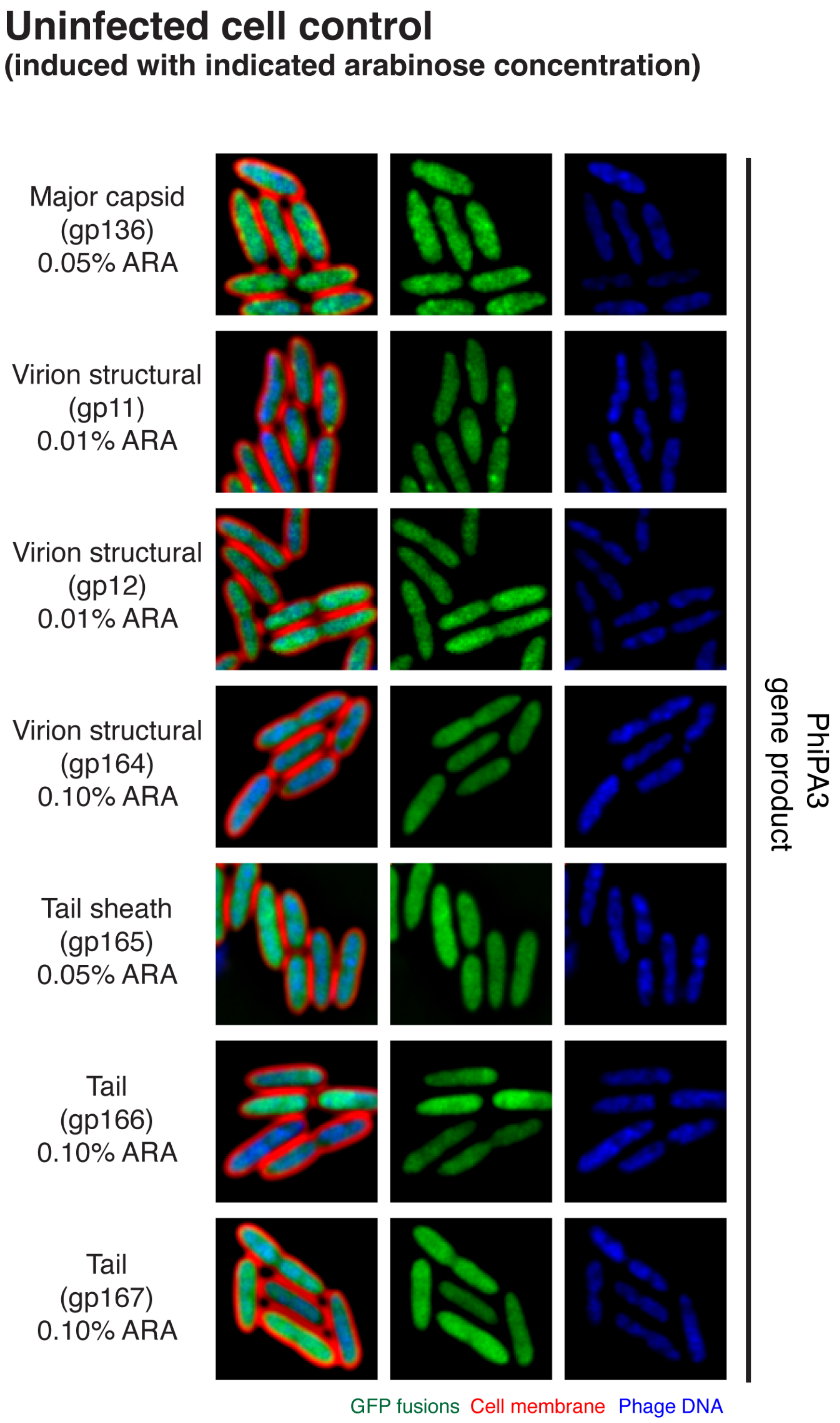

Fig. S1. Fluorescent micrographs of uninfected control cells. The cells were grown on agarose pads, incubated at 30°C for 3 hours, and were induced by indicated arabinose concentration to express fluorescent protein fusions. Membranes were stained with FM 4-64 (red) and DNA with DAPI (blue). Cover slips were put on immediately before the microcopy and images were then collected. PA3-gp12, PA3-gp136, PA3-gp164, PA3- gp165, PA3-gp166, PA3-gp167 appeared uniformly distributed throughout the cells in the absence of infection whereas PA3-gp11 formed small foci. Scale bar, 1 micron.

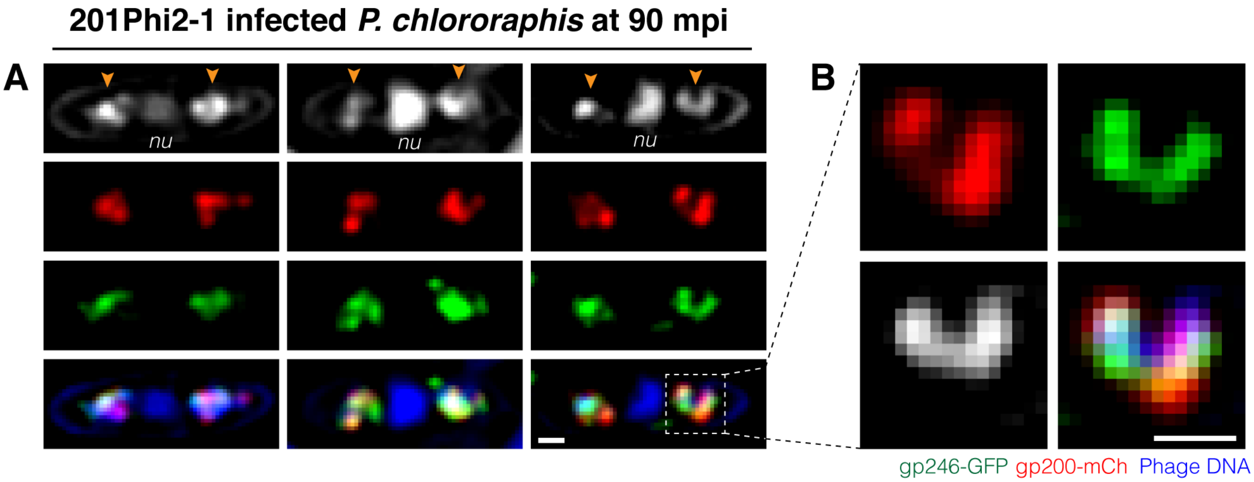

Fig. S2. Fluorescent micrographs of 201Phi2-1 infected *P. chlororaphis* cells expressing major capsid (gp200)-mCherry and internal head protein (gp246)-GFP showing phage maturation of phage 201Phi2-1 *in vivo*. (A, B). At late time point after DNA packaging, extranuclear staining structures we term "Phage Bouquet" (orange arrows) appear adjacent to the phage nucleus (nu). Major capsid (gp200, red) and the internal head protein (gp246, green) colocalize on the structure suggesting that the structures are comprised of the DNA packaged-mature capsids. Scale bar, 1 micron. The region inside the dashed box in (A) is magnified in (B) to more clearly show the colocalization of major capsid and the internal head protein on the phage bouquet. DAPI staining is shown in white in the top row panel A, in blue in the bottom row of panel A, and in white and blue in panel B. Scale bar, 0.5 micron.

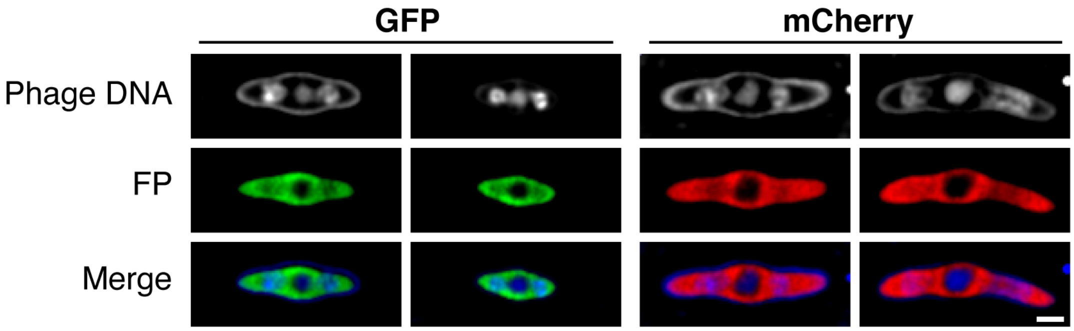

Fig. S3. Fluorescent micrographs of PA3 infected *P. aeruginosa* cells expressing fluorescent protein; GFP and mCherry. The cells were grown on agarose pads and were induced by 0.2% arabinose to express the fluorescent protein prior to infection. At late time point, phage bouquets were assembled and partially excluded fluorescent proteins suggesting that the physiology inside phage bouquet is distinct from cell cytoplasm. Scale bar, 1 micron.

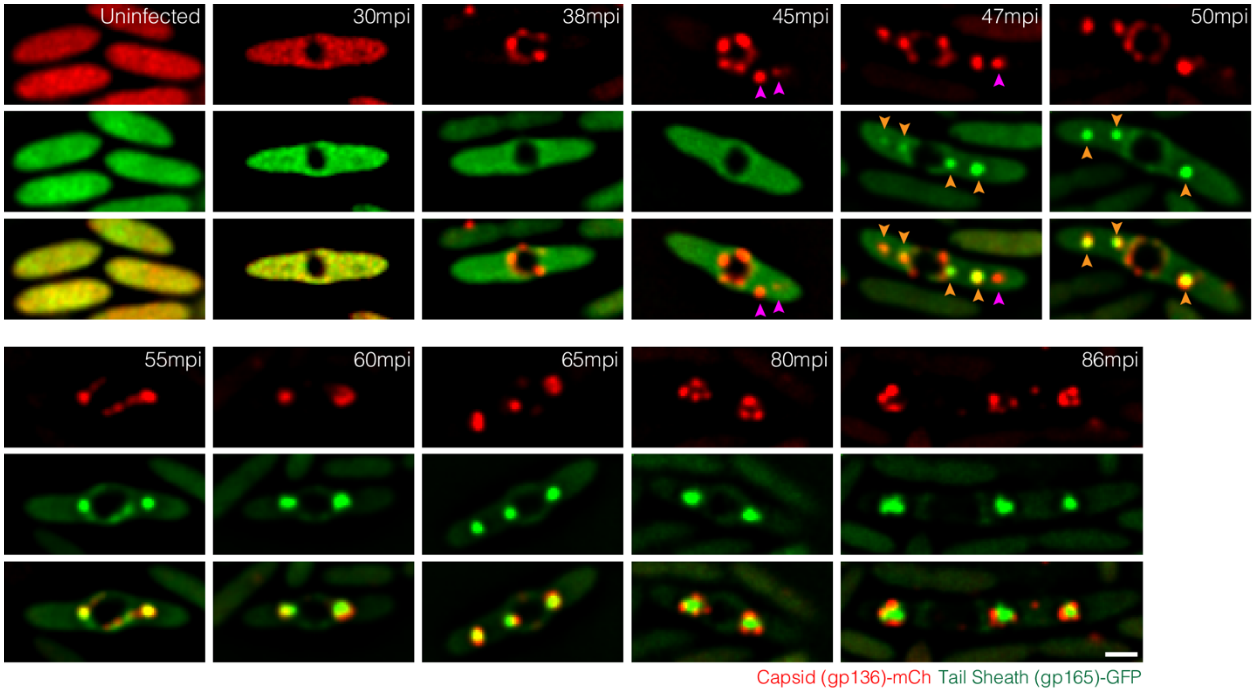

Fig. S4. Time-series of fluorescent micrographs of PA3 infected *P. aeruginosa* cells expressing major capsid (gp136)-mCherry and tail sheath (gp165)-GFP. The cells were grown on agarose pads and were induced by 0.05% arabinose to express fluorescent proteins. High titer phage PA3 lysates were added on top of the pad to initiate infection. Time-series throughout infection were imaged and collected. By 45 mpi, capsids assemble, package DNA at the phage nucleus, detach and localize in the cytoplasm. At this time point, capsids (red foci) did not appear to contain assembled tails (pink arrows). After 45 mpi, more capsids were incorporated into phage bouquets where tails localized inside and were surrounded by capsids (orange arrows). Scale bar, 1 micron.

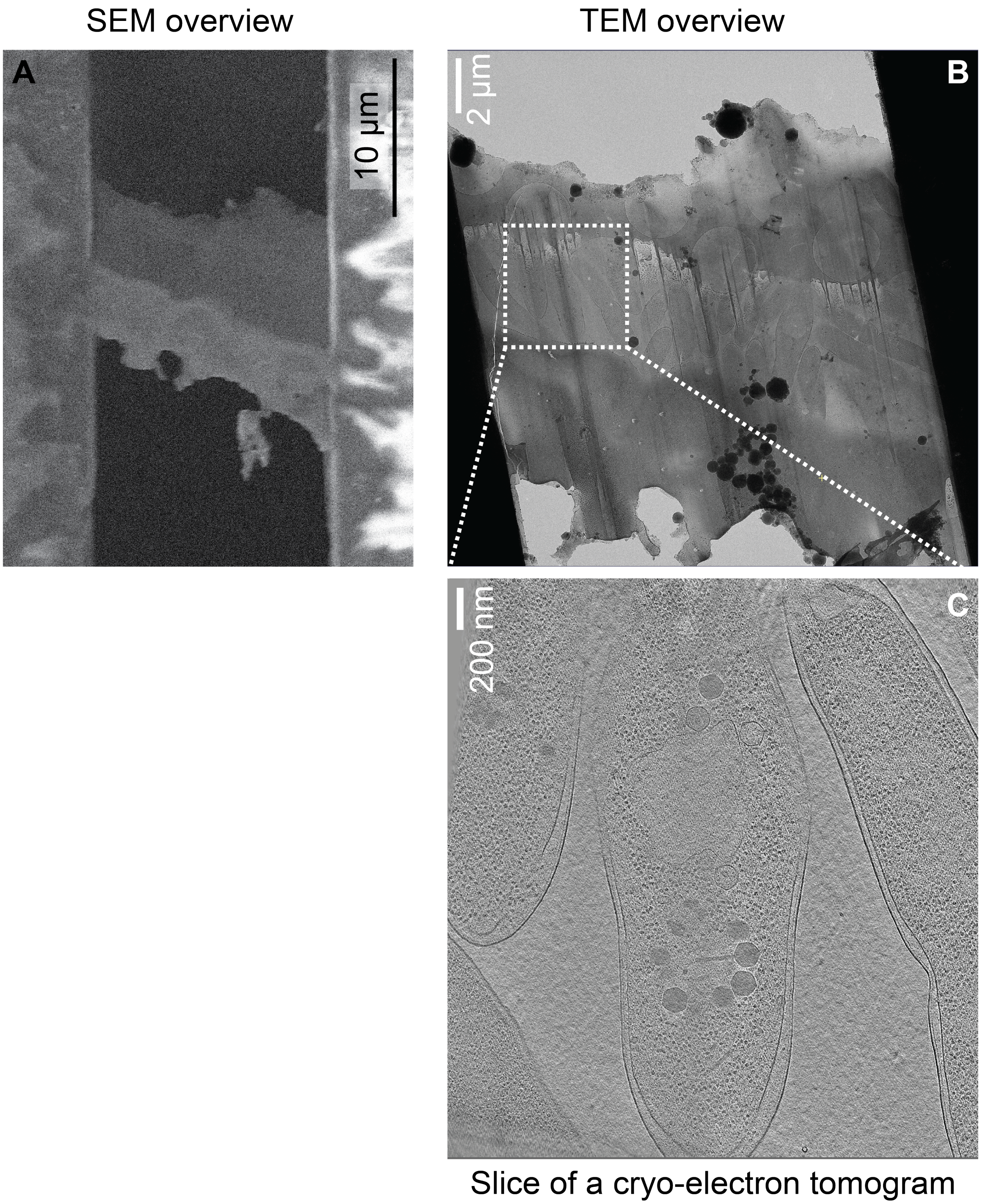

Fig. S5. Overview of lamella in Scanning Electron Microscopy (SEM) view and in Transmission Electron Microscopy (TEM) corresponding to the tomogram acquired in Figure 4. Scale bars: 10 µm (SEM), 2 µm (TEM), 200 nm (tomogram slice).

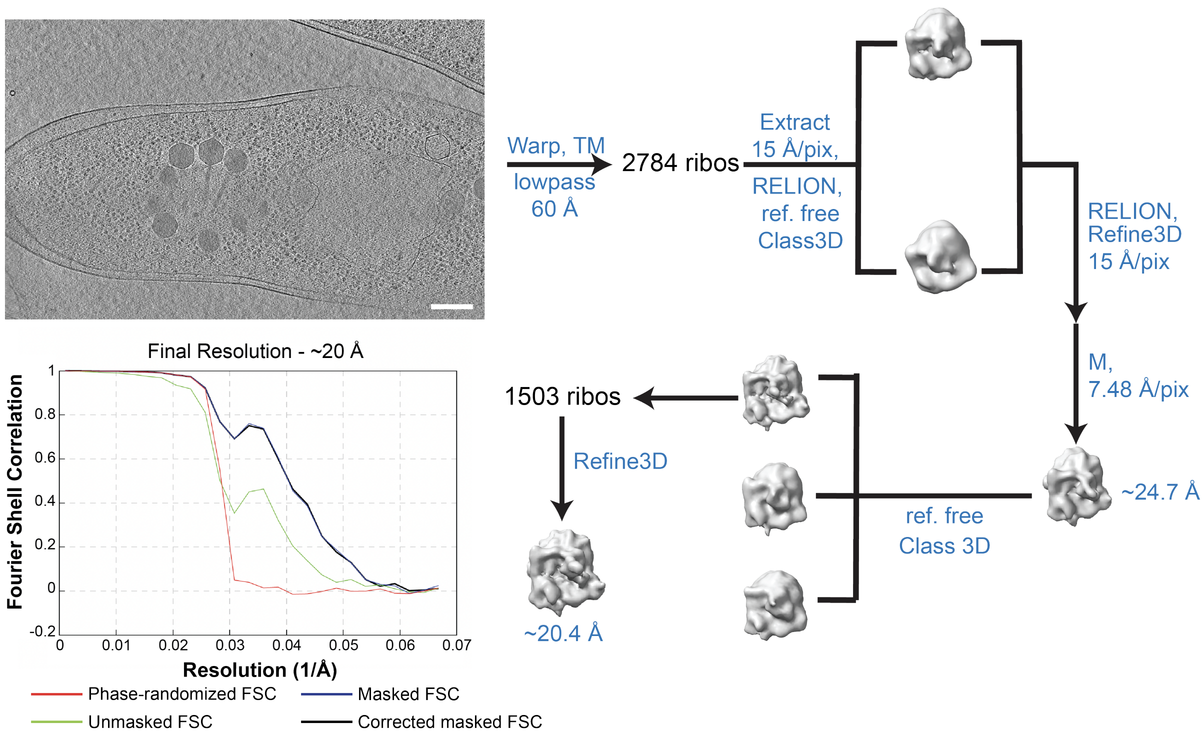

Fig. S6. Pipeline showing the methodolgy to obtain ribosome average from the tomogram shown in Figure 4. 3-dimensional template matching (TM) was performed in Warp *(50)* and the extracted ribosomes were passed through 3D classification and 3D refinement operations in RELION *(51)* and further refinement in M *(52)*. Scale bar for the tomogram: 200 nm.

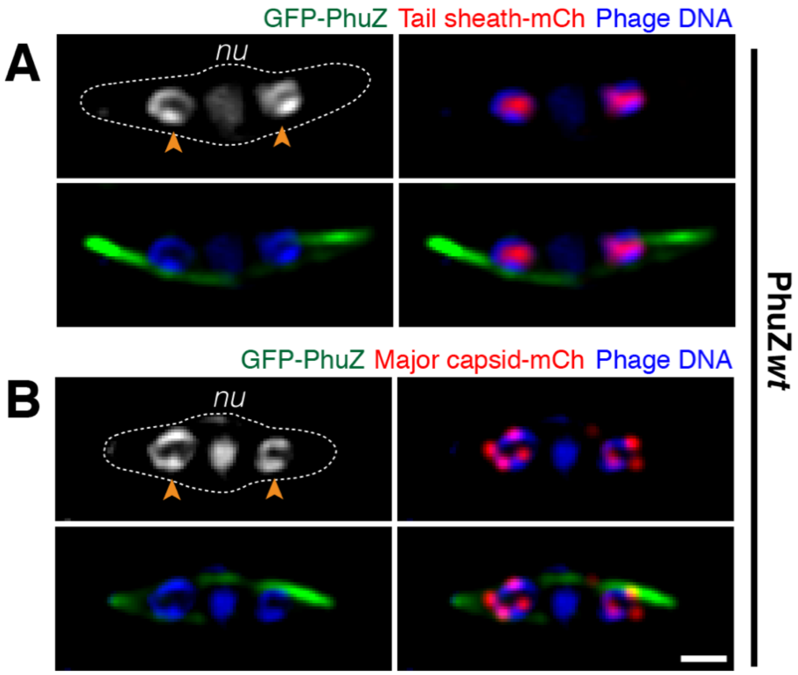

Fig. S7. Fluorescent micrographs of PA3 infected *P. aeruginosa* cells expressing wildtype GFP-PhuZ with either tail sheath (gp165)-mCherry (A) or major capsid (gp136)- mCherry (B) at late infection. The cells were grown on agarose pads and were induced by 0.025% arabinose to express the fluorescent proteins. High titer phage PA3 lysates were added on top of the pad to initiate infection. In the presence wildtype PhuZ filament, phage bouquets were located adjacent to the phage nucleus. Major capsids and tail sheath were localized and associated with the phage bouquets. Scale bar, 1 micron.

Table S1. Plasmids and strains used in this study

Description (DNA insert/Vector)	Plasmid	Strain	Phage[a]	Host[b]	Source
gp11-GFP / pHERD-30T	pMAC091	MAC075	PA3	K2733	This study
gp12-GFP / pHERD-30T	pMAC066	MAC072	PA3	K2733	This study
gp136-GFP / pHERD-30T	pVC87	VC454	PA3	K2733	This study
gp164-GFP / pHERD-30T	pKN67	KN204	PA3	K2733	This study
gp165-GFP / pHERD-30T	pKN105	VC456	PA3	K2733	This study
gp166-GFP / pHERD-30T	pKN051	KN205	PA3	K2733	This study
gp167-GFP / pHERD-30T	pKN037	KN206	PA3	K2733	This study
gp136-GFP-mCh-gp053 / pHERD-30T	pVC101	VC489	PA3	K2733	This study
gp165-GFP-mCh-gp053 / pHERD-30T	pVC102	VC491	PA3	K2733	This study
gp136-mCh-gp165-GFP / pHERD-30T	pVC95	VC465	PA3	K2733	This study
GFP-PhuZ	pME056	VC568	PA3	K2733	This study
GFP-PhuZD190A	pVC032	VC569	PA3	K2733	This study
gp136-mCh-GFP-PhuZ	pVC103	VC494	PA3	K2733	This study
gp136-mCh-GFP-PhuZD190A	pVC104	VC496	PA3	K2733	This study
gp165-mCh-GFP-PhuZ	pVC114	VC572	PA3	K2733	This study
gp165-mCh-GFP-PhuZD190A	pVC115	VC574	PA3	K2733	This study
Host 50sL28-GFP / pHERD-30T	pKN057	MAC128	-	K2733	This study
Host thymidylate kinase (TMK)-GFP / pHERD-30T	pMAC120	MAC122	-	K2733	This study
Host thymidylate synthase (TMS)-GFP / pHERD-30T	pMAC121	MAC123	-	K2733	This study
gp30-GFP / pHERD-30T	pMAC001	MAC003	201	PC	This study
gp246-GFP-gp200-mch / pHERD-30T	pVC94	VC447	201	PC	Chaikeeratisak et al., 2019 *(21)*
mCh / pHERD-30T	KN157	VC529	-	PA01	Chaikeeratisak et al., 2017 *(12)*
GFP / pHERD-30T	pKN151	VC530	-	PA01	Chaikeeratisak et al., 2017 *(12)*

[a]201, *P. chlororaphis* phage 201phi2-1. PA3, *P. aeruginosa* phage PhiPA3

[b]PC, *P. chlororaphis*. PA01, *P. aeruginosa*. K2733 is a PA01 derivative lacking multiple efflux pumps (Δ*mexB*, Δ*mexX*, *ΔmexCD-oprJ*, Δ*mexEF-oprN*) and was used to allow better staining with fluorescent dyes. Identical results were obtained in both PA01 and K2733.

Supplemental Movie Legends

Movie S1. Series of slices through a cryo-electron tomogram of phage PhiPA3-infected *P. aeruginosa* at 75 mpi (see Figure 4). Scale bar: 200 nm.

Movie S2. Additional example showing series of slices through a cryo-electron tomogram of phage PhiPA3-infected *P. aeruginosa* at 75 mpi. Scale bar: 200 nm.

Movie S3. Additional example showing series of slices through a cryo-electron tomogram of phage PhiPA3-infected *P. aeruginosa* at 75 mpi. Scale bar: 200 nm.

Movie S4. Additional example showing series of slices through a cryo-electron tomogram of phage PhiPA3-infected *P. aeruginosa* at 75 mpi. Scale bar: 200 nm.
